# Effects of Exercise Intervention (with and without Joint Mobilization) in Patients with Adhesive Capsulitis: A Systematic Review and Meta-Analysis

**DOI:** 10.3390/healthcare11101504

**Published:** 2023-05-22

**Authors:** Jong Hyeon Lee, Hyung Gyu Jeon, Yong Jin Yoon

**Affiliations:** 1 Department of Sport Industry Studies, Yonsei University, Seoul 03722, Republic of Korea; leejh01@yonsei.ac.kr; 2 Department of Physical Education, Yonsei University, Seoul 03722, Republic of Korea; hgjeon@yonsei.ac.kr; 3 International Olympic Committee Research Centre KOREA, Yonsei University, Seoul 03722, Republic of Korea

**Keywords:** adhesive capsulitis, frozen shoulder, glenohumeral joint, exercise, joint mobilization, meta-analysis

## Abstract

This review aimed to investigate the effects of exercise and exercise with joint mobilization on shoulder range of motion (ROM) and subjective symptom recovery in patients with adhesive capsulitis (AC). Related Studies published from 2000 to 2021 that were peer-reviewed and for which pre-and post-values could be calculated were extracted from PubMed, CINAHL, SPORTDiscus, and Web of Science. Nine studies met our inclusion criteria. As a result of calculating the standard mean difference (SMD) and 95% confidence intervals (CI), both exercise and exercise with joint mobilization showed a large effect on shoulder ROM and subjective outcomes. The combination showed a more significant effect than exercise alone on shoulder flexion (SMD = −1.59 [−2.34, −0.65]), extension (SMD = −1.47 [−2.05, −0.89]), internal rotation (SMD = −1.77 [−2.17, −1.36], external rotation (SMD = −2.18 [−2.92, −1.44]), and abduction ROM (SMD = −1.99 [CI −3.86, −0.12]). Patients who performed exercise alone showed a higher effect of improvement in subjective function (SMD = 3.15 [2.06, 4.24]) and pain (SMD = 4.13 [1.86, 6.41]). Based on these results, an AC rehabilitation exercise program should be developed by adjusting the amount of exercise and joint mobilization by identifying the patient’s needs, subjective symptoms, and ROM.

## 1. Introduction

Adhesive capsulitis (AC), also referred to as frozen shoulder, involves progressive thickening and shrinkage of the glenohumeral joint capsule [1,2]. Approximately 2–5% of the world’s population experiences AC [3]. AC occurs more commonly in women aged 40–60 years and in sedentary workers than in manual workers [4]. AC progresses through the pre-adhesion, acute adhesive, maturation, and chronic stages [4], and symptoms vary according to the stage. Nocturnal pain occurs in the pre-adhesion stage, and there is no decrease in the shoulder range of motion (ROM); however, a loss of motion begins to appear in the acute adhesive stage. Pain decreases in the maturation stage compared to that in the previous stage within the ROM; however, the motion is further reduced as it reaches the chronic stage when adhesions have fully progressed, and pain occurs when arm movement exceeds the restricted ROM [5]. In particular, a prolonged period of limited movement of the affected shoulder can cause weakening of muscles, tendons, ligament contraction [6], impaired muscle coactivation, and proprioception of the shoulder [7]. In addition, due to thickening of the coracohumeral ligament, external rotation is limited, and, as it also affects the subscapular and supraspinatus tendons, internal rotation can also be restricted [8,9,10]. If AC is left unattended and progresses to more advanced stages, the thickening and contraction of the glenohumeral capsule ends occurs, with limited shoulder ROM in all directions [11,12]. As such, decreased ROM accompanied by pain reduces the quality of life by restricting daily living activities, such as washing the back, making the bed, pulling the car seat belt, working, and several leisure activities [13,14,15]. Therefore, recovery through appropriate intervention is necessary.

It is essential to design movements to alleviate symptoms in patients with AC. Exercise and manual therapy approaches, such as joint mobilization [16,17], have been used to reduce pain and improve patients’ ROM and function. Exercises designed to improve function and ROM can improve shoulder joint stability, mobility, and proprioception [18]. In a systematic review examining the effect of physiotherapy for AC [2], stretching along with administration of corticosteroid injection, physical therapy, and other modalities was effective in relieving pain and improving ROM and shoulder function in patients with stage 2–3 AC. Meanwhile, in a meta-analysis of the effect of joint mobilization on the recovery of shoulder ROM, conducting joint mobilization yielded an increase in ROM by 20.14° greater than that of the control group with respect to shoulder abduction and external rotation. Furthermore, a medium effect was observed, suggesting that joint mobilization was effective in restoring ROM and relieving pain [19]. In addition, mobilization of peripheral somatosensory receptors and suppression of nociceptors resulted in decreased pain and increased shoulder mobility by enhancing the exchange between synovial fluid and cartilage [20,21]. Thus, exercise and mobilization could be effective interventions in patients with AC.

However, as indicated in a few studies, the exercise and joint mobilization results for AC are contradictory to those reported in previous studies. For example, [22] indicated that stretching with glenohumeral joint distraction and glide toward caudal, posterior, and anterior directions significantly improved the range of shoulder flexion and abduction in patients with AC compared to stretching alone. In contrast, [23] reported decreased pain but no significant group difference between exercise and exercise with Maitland mobilization technique in grade II and III. Likewise, posterior gliding improves shoulder ROM in all directions except abduction, and the mechanism of elevation of the humeral head and the increase in the thickness of the inferior fibers of the joint capsule has been elucidated, but, compared to the control group, a significant increase in abduction ROM of 20.14 degrees has also been reported [21,24,25]. Therefore, joint mobilization may be effective for ROM recovery and pain reduction, but several studies have revealed that the evidence is not conclusive [19]. These conflicting results suggest the importance of establishing an evidence-based decision on whether to intervene only with exercise or to add joint mobilization.

Because exercise can be easily performed in daily life, it is desirable to prevent financial waste by judging when joint mobilization is unnecessary. Therefore, to make efficient clinical decisions, analysis of the effects of exercise alone versus exercise combined with other interventions is essential.

This review aimed to present a comprehensive conclusion considering AC patients’ objective indicators, such as shoulder ROM, subjective function measured by a questionnaire, and degree of pain. In addition, this study aimed to present a more generalized conclusion on the impact of an exercise intervention on symptom improvement by analyzing previous studies on the effects of exercise and joint mobilization in patients with AC. By comparing the effects of exercise and exercise plus joint mobilization, implications for efficient intervention in the field of exercise prescription can be presented.

## 2. Methods

A systematic search was conducted to investigate the effects of exercise and a combination of exercise and joint mobilization in patients with AC according to the Preferred Reporting Items for Systematic Review and Meta-Analysis (PRISMA) guidelines [26]. In this review, the following research questions were set: 1. Investigation of the effect of exercise and exercise with joint mobilization on shoulder ROM and subjective function in AC patients. 2. Comparison of effects according to whether or not joint mobilization is applied to exercise.

### 2.1. Literature Search Strategy

A comprehensive literature search was performed to identify peer-reviewed articles. We systematically searched PubMed, CINAHL, SPORTDiscus, and Web of Science electronic databases for papers published from January 2000 to November 2021. The period of collection and screening of papers was from June to November 2021. We used a keyword search and the medical subject headings vocabulary. The search was limited to studies involving humans, written in English, and reported in peer-reviewed journals. The search terms were as follows: adhesive capsulitis, frozen shoulder, frozen shoulders, adhesive capsulitides, shoulder, periarthritis, stiff, shoulder stiffness, contracted, restricted, peri capsulitis, shoulder pain, fibrosis, irritative, AND exercise, sport, sports, training, stretching, rehabilitation, intervention; NOT calcific tendinitis, rotator cuff tear, rotator cuff rupture, SLAP, labral tear, labral rupture, biceps tendinitis, Bankart, impingement, tear, and rupture. We limited the scope of the search to the case where the full text was retrieved from each database. In addition, while using PubMed, we searched by setting “Article Type” to “Randomized controlled trial” and “Clinical Trial”, and “Books and Documents”, “Meta-Analysis”, “Review”, and “Systematic review” set to exclude. In CINAHL and SPORTDiscus, we set “Source Types” to “Academic Journals”, in Web of Science we set “Document Types” to “Articles”, and in “Quick Filters” we set “Review Article” and “Early Access” to exclude. A manual search for relevant references was performed on all systematically retrieved studies, and the identified articles were screened by two independent authors (J.H.L. and H.G.J) who specialize in sports rehabilitation.

### 2.2. Eligibility Criteria

The inclusion and exclusion criteria described in the following paragraphs were assessed by the investigators using the eligibility of the articles identified in the systematic search.

#### 2.2.1. Inclusion Criteria

The inclusion criteria used to select and screen studies were as follows:

- A peer-reviewed study published in English between 2000 and 2021 investigating the effect of exercise and joint mobilization in patients with AC;
- Outcome variables including shoulder ROM, subjective function, and pain;
- Studies in which the results were described or converted to mean and standard deviation.

#### 2.2.2. Exclusion Criteria

The exclusion criteria used to screen out studies were as follows:

- Case studies using a single-subject design;
- Studies that did not definitively establish that the participants were patients with AC;
- Studies in which interventions other than exercise and/or exercise with joint mobilization were added and applied as confounding variables (e.g., injection, operation, drug, ultrasound).

### 2.3. Assessment of Methodologic Quality

The authors assessed the quality of included studies using the National Heart, Lung, and Blood Institute Quality Assessment Tool (NHLBI-QAT, https://www.nhlbi.nih.gov/health-topics/study-quality-assessment-tools, accessed on 15 February 2022). The checklist includes 11 questions and indicates the total score as a percentage. Two authors independently reviewed the full text of the selected studies for quality analysis. Discrepancies in screening and scoring were addressed through collaboration between the authors until a consensus was reached. If a consensus could not be reached, any conflicts of opinion were resolved by a third reviewer (corresponding author of this review who is an expert in meta-analysis and other research methodologies). The screened studies were classified into levels 1–3 according to the study quality. In this present review, NHLBI scores ≥ 70%, 40% < NHLBI scores < 70%, and ≤40% were considered levels of evidence 1, 2, and 3, respectively.

### 2.4. Assessment of Publication Bias

After reviewing the meta-analysis data using a forest plot, the asymmetry of the effect size was visually assessed using a funnel plot. In addition, the relationship between effect size and standard error was verified using Egger’s regression to determine whether the funnel plot was asymmetric. In the case of asymmetry, we calculated the average effect size obtained by adjusting the asymmetry using the trim-and-fill method and compared it with the original average effect size [27].

### 2.5. Data Synthesis and Extraction

To calculate the standard mean difference (SMD) and 95% confidence intervals (CIs), means, standard deviations pre-and post-intervention, and numbers were extracted from each study. The review process was performed by assessing the aims and quality of the studies, characteristics, inclusion and exclusion criteria of the subjects, intervention procedures, and outcome variables. The significance of heterogeneity varied depending on the I^2^ value: high (≥75%), medium (≥25%, <75%), and low (<25%). A random-effects model was used in the meta-analysis to generalize the results of the independent studies. We used R-Studio software (Version 4.0.2, R-Studio, Boston, MA, USA) with the “metafor” package to calculate SMD and assess publication bias through forest and funnel plots. Cohen’s d was used to calculate and determine the effect size [28]; the CIs for the effect size was 95%, and the significance level was 0.05. A fixed-effects model was used to estimate the overall effect when homogeneity test statistics were insignificant. When the heterogeneity was *p* ≤ 0.05, a random-effects model was used, including the restricted maximum likelihood estimation method.

## 3. Results

### 3.1. Study Selection

**Figure 1:**
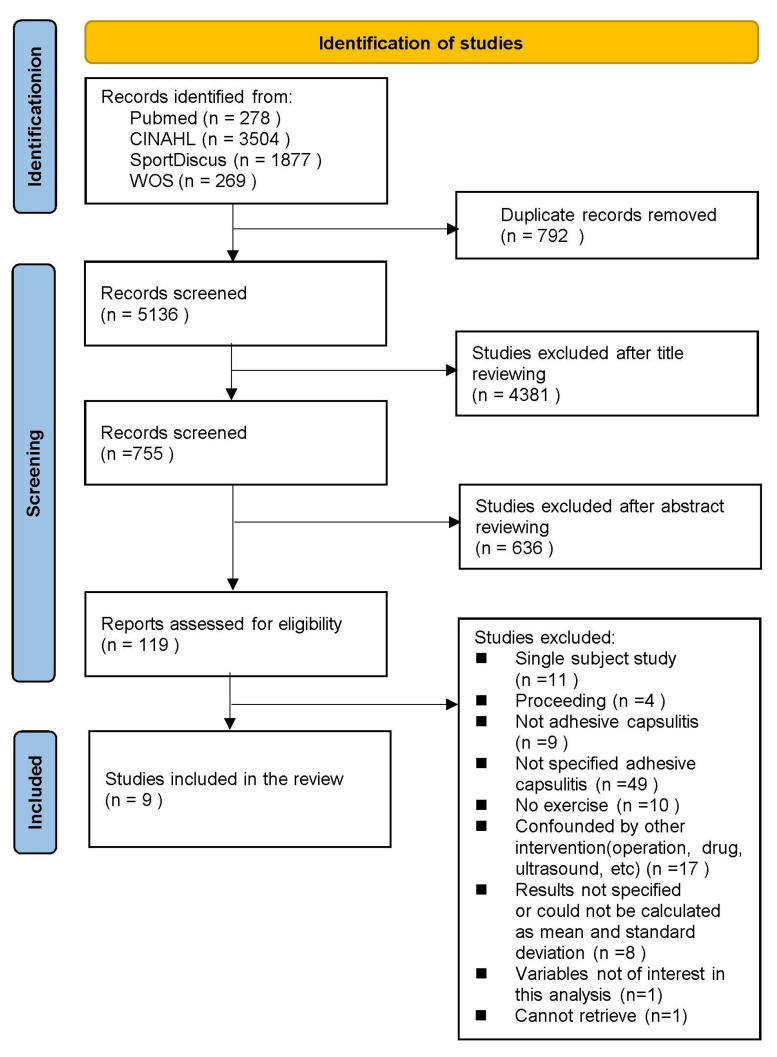
Flow chart of the selection process for studies.

The flowchart presented in Figure 1 follows the PRISMA guidelines. The first search identified 5136 relevant studies. After screening, nine studies were included in the meta-analysis. However, no additional studies were identified after examining the references of the pooled studies. Table 1 and Table 2 present methodological summaries of the included studies.

### 3.2. Level of Evidence and Strength of Recommendation

The average methodological quality of the included studies was 8.8 out of a possible 11 (range 6–10; Table 3). Seven studies [22,29,31,32,34,35,36] were classified as Level 1 with an average NHLBI score of 9.4, while 2 studies [30,33] were classified as Level 2 with an average NHLBI score of 6.5 (Table 3). Regarding grading according to variables, grade A was assigned when consistent results were obtained in most pooled studies with high-quality RCT designs. Grade B was assigned if most of the studies included in each variable had case-control or comparative experimental research designs. Therefore, only the pain variable was classified as grade A based on the effects of exercise. In contrast, flexion, extension, internal rotation, external rotation, abduction ROM, and subjective function variables were classified as grade B. The effects of a combination of exercise and joint mobilization, flexion, extension, internal rotation, external rotation ROM, and pain variables were graded as A, whereas abduction ROM and subjective function were graded as B.

### 3.3. Publication Bias

**Figure 2:**
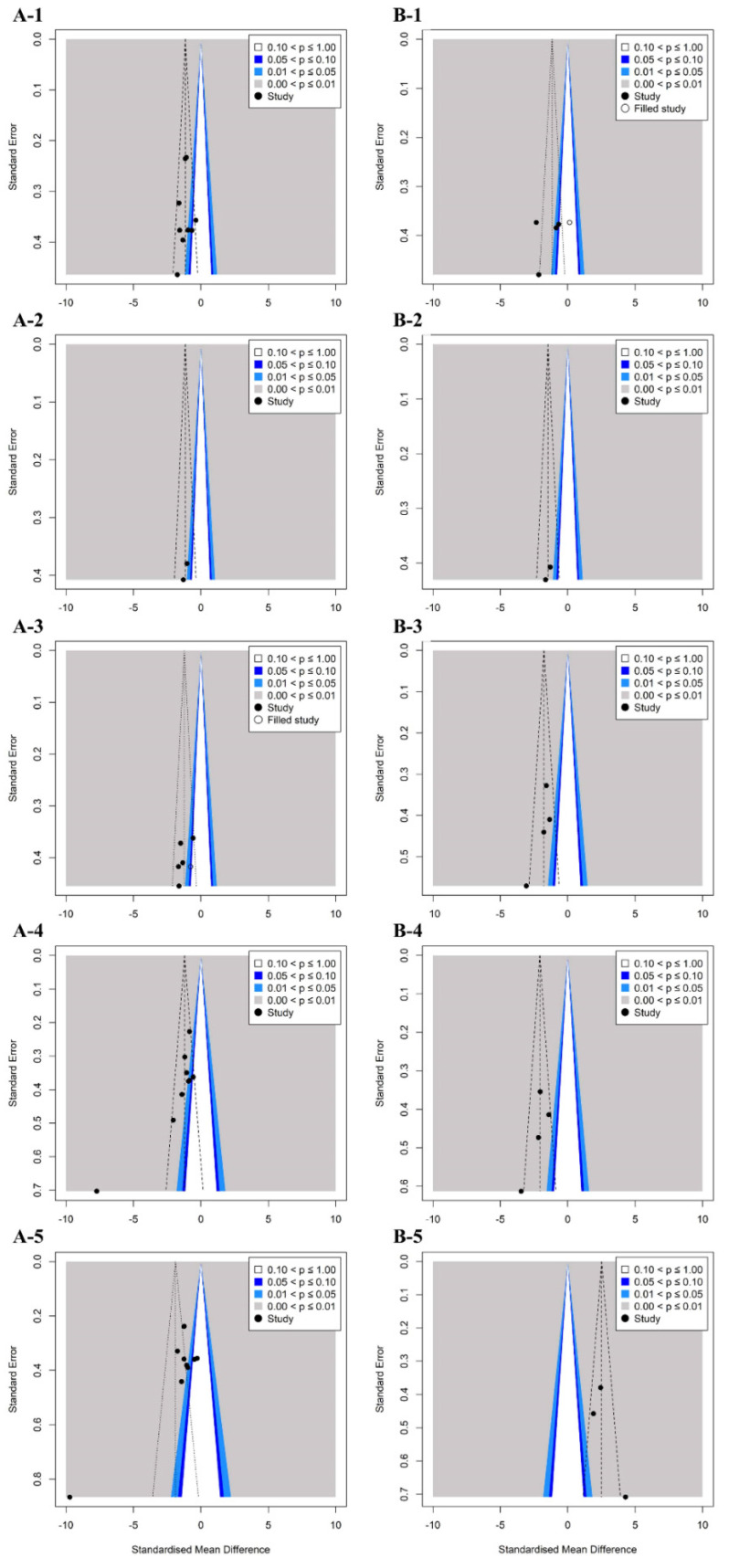
Funnel plots of standard error by each shoulder range of motion for the studies included in the meta-analysis. Funnel plots of exercise effect on flexion (**A-1**), extension (**A-2**), internal rotation (**A-3**), external rotation (**A-4**), and abduction range of motion (**A-5**). Funnel plots combination effect on flexion (**B-1**), extension (**B-2**), internal rotation (**B-3**), external rotation (**B-4**), and abduction range of motion (**B-5**).

**Figure 3:**
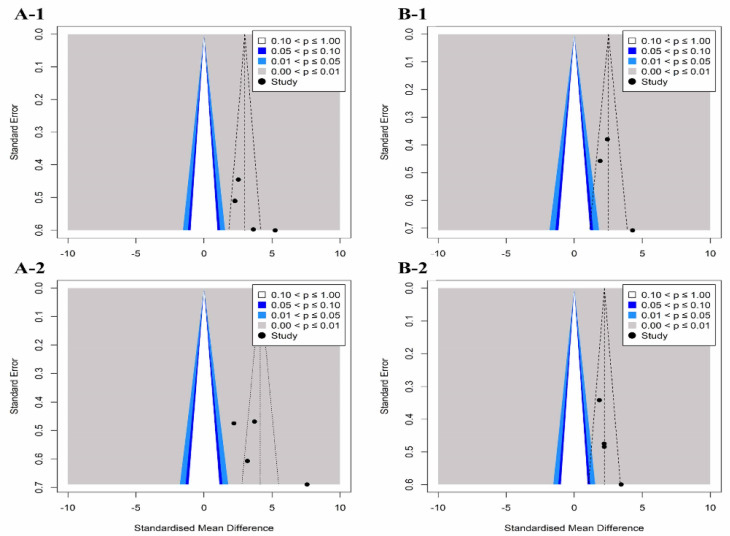
Funnel plots of standard error by each patient-oriented outcome for the studies included in the meta-analysis. Funnel plots of exercise effect on subjective function (**A-1**) and pain (**A-2**). Funnel plots of combination effect on subjective function (**B-1**) and pain (**B-2**).

Egger’s regression analysis, which was conducted to determine the symmetry of the funnel plot (Figure 2 and Figure 3), confirmed that flexion (*p* = 0.14), extension (*p* = 0.62), and internal rotation (*p* = 0.24) ROM variables in studies investigating the effects of exercise showed symmetry, indicating no publication error.

In studies exploring the effects of a combination of exercise and joint mobilization, extension (*p* = 0.56), internal rotation (*p* = 0.07), ROM, and pain (*p* = 0.12) had no publication errors.

In contrast, in studies investigating the effects of exercise, asymmetry of external rotation (*p* < 0.01), abduction ROM (*p* < 0.01), subjective function (*p* < 0.01), and pain (*p* < 0.01) were noted, and publication errors were confirmed. Studies exploring the effects of a combination of exercise and joint mobilization, flexion and abduction ROM, and subjective function variables have shown asymmetry and publication errors. Therefore, we applied the trim-and-fill method to these variables and confirmed the effect of publication errors on the research results. When comparing the existing SMD with the adjusted SMD, the difference was <10%. Therefore, it can be concluded that publication errors had less effect on the results of this study.

### 3.4. Data Synthesis

**Figure 4:**
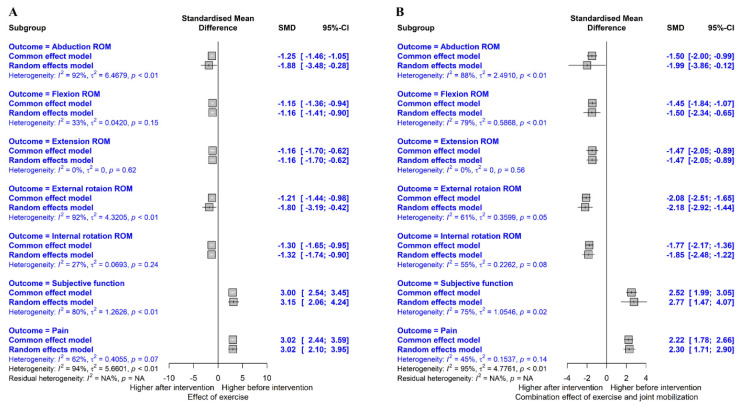
Summary of meta-analysis results. Effect of exercise (**A**) and combination effect of exercise and joint mobilization (**B**).

The analysis effect size showed that both exercise and exercise combined with joint mobilization showed a large effect on shoulder ROM, subjective function, and pain recovery. The pooled effect size for each variable is summarized in Figure 4. 

#### 3.4.1. Flexion ROM

**Figure 5:**
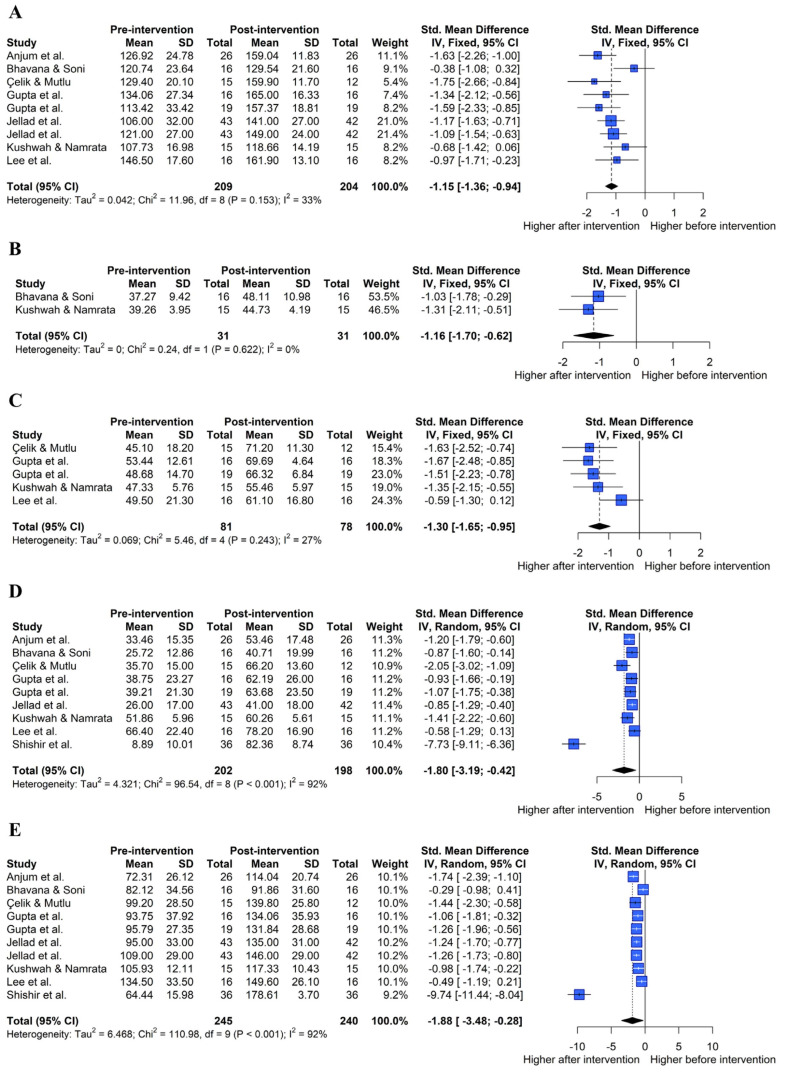
Effect of exercise on shoulder range of motion in forest plots of the meta-analysis. Flexion (**A**), extension (**B**), internal rotation (**C**), external rotation (**D**), and abduction range of motion (**E**) [22,29,30,31,32,33,34,36].

Figure 5A shows the overall effect size measures for the effect of exercise on flexion ROM in patients with AC using a forest plot (k = 9, Q(8) = 11.96, p = 0.153, I^2^ = 33%). Under the fixed-effects model, the overall difference in exercise on flexion ROM was statistically significant (SMD = −1.15, 95% CIs = −1.36 to −0.94), indicating that exercise significantly improved flexion ROM in patients with AC compared to pre-exercise (large effect size).

**Figure 6:**
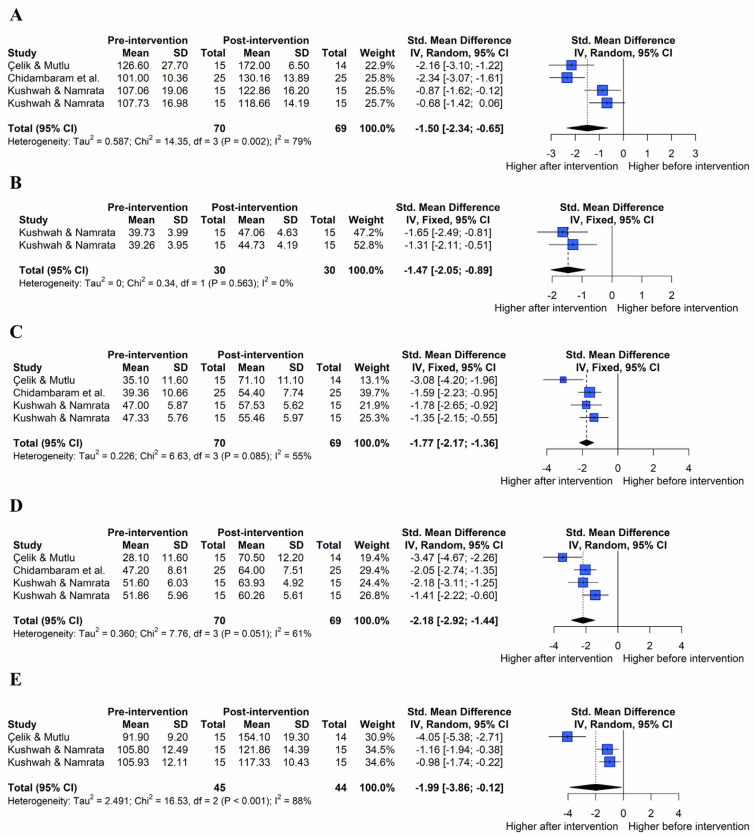
Effect of a combination of exercise and joint mobilization on shoulder range of motion in forest plots of the meta-analysis. Flexion (**A**), extension (**B**), internal rotation (**C**), external rotation (**D**), and abduction range of motion (**E**) [22,35,36].

The effect of the combination of exercise and joint mobilization on flexion ROM was assessed (Figure 6A; k = 4, Q(3) = 14.35, *p* = 0.002, I^2^ = 79%). Under the random-effects model, the overall effect of a combination of exercise and joint mobilization on flexion ROM was statistically significant (SMD = −1.50, 95% CIs = −2.34 to −0.65), indicating that a combination of the two interventions led to the improvement of flexion ROM in patients with AC compared with pre-intervention (large effect size).

#### 3.4.2. Extension ROM

Figure 5B shows the overall effect size measures of the effect of exercise on extension ROM in patients with AC (k = 2, Q(1) = 0.24, *p* = 0.622, I^2^ = 0%). Under the fixed-effects model, the overall difference in exercise on extension ROM was statistically significant (SMD = −1.16, 95% Cis = −1.70 to −0.62), indicating that exercise led to the improvement of extension ROM in patients with AC compared to pre-exercise (large effect size).

The effect of the combination of exercise and joint mobilization on extension ROM was assessed (Figure 6B; k = 2, Q(1) = 0.34, *p* = 0.563, I^2^ = 0%). Under the fixed-effects model, the overall effect of a combination of exercise and joint mobilization on extension ROM was statistically significant (SMD = −1.47, 95% CIs = −2.05 to −0.89), indicating that a combination of the two interventions improved extension ROM in patients with AC compared with pre-intervention (large effect size).

#### 3.4.3. Internal Rotation ROM

Figure 5C shows the overall effect size measures of the effect of exercise on internal rotation ROM in patients with AC (k = 5, Q(4) = 5.46, *p* = 0.243, I^2^ = 27%). Under the fixed-effects model, the overall difference in exercise on internal rotation ROM was statistically significant (SMD = −1.30, 95% CIs = −1.65 to −0.95), indicating that exercise improved the internal rotation ROM in patients with AC compared to pre-exercise (large effect size).

The effect of the combination of exercise and joint mobilization on internal rotation ROM was assessed (Figure 6C; k = 4, Q(3) = 6.63, *p* = 0.085, I^2^ = 55%). Under the fixed-effects model, the overall effect of a combination of exercise and joint mobilization on internal rotation ROM was statistically significant (SMD = −1.77, 95% CIs = −2.17 to −1.36), indicating that a combination of the two interventions improved internal rotation ROM in patients with AC compared with pre-intervention (large effect size).

#### 3.4.4. External Rotation ROM

Figure 5D shows the overall effect size measures for the effect of exercise on external rotation ROM in patients with AC (k = 9, Q(8) = 96.54, *p* < 0.001, I^2^ = 92%). Under the random-effects model, the overall difference in exercise on external rotation ROM was statistically significant (SMD = −1.80, 95% CIs = −3.19, −0.42), indicating that exercise improved external rotation ROM in patients with AC compared to pre-exercise (large effect size).

The effect of the combination of exercise and joint mobilization on external rotation ROM was assessed (Figure 6D; k = 4, Q(3) = 7.76, *p* = 0.051, I^2^ = 61%). Under the random-effects model, the overall effect of the combined exercise and joint mobilization on external rotation ROM was statistically significant (SMD = −2.18, 95% CIs = −2.92 to −1.44), indicating that a combination of the two interventions improved external rotation ROM in patients with AC compared with pre-intervention (large effect size).

#### 3.4.5. Abduction ROM

Figure 5E shows the overall effect size measures of the effect of exercise on abduction ROM in patients with AC (k = 10, Q(9) = 110.98, *p* < 0.001, I^2^ = 92%). Under the random-effects model, the overall difference in exercise on abduction ROM was statistically significant (SMD = −1.88, 95% CIs = −3.48, −0.28), indicating that exercise improved abduction ROM in patients with AC compared to pre-exercise (large effect size).

The effect of the combination of exercise and joint mobilization on abduction ROM was assessed (Figure 6E; k = 3, Q(2) = 16.53, *p* < 0.001, I^2^ = 88%). Under the random-effects model, the overall effect of a combination exercise and joint mobilization on abduction ROM was statistically significant (SMD = −1.99, 95% CIs = −3.86, −0.12), indicating that a combination of the two interventions improved abduction ROM in patients with AC compared to pre-intervention (large effect size).

#### 3.4.6. Subjective Function

**Figure 7:**
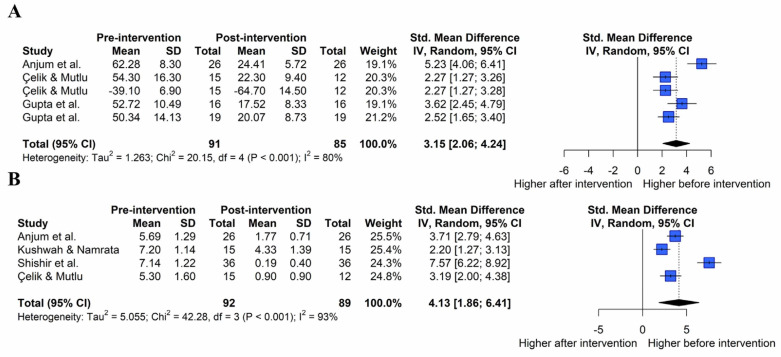
Effect of exercise on patient-oriented outcome in forest plots of the meta-analysis. Subjective function (**A**) and pain (**B**) [22,29,31,34,36].

Figure 7A shows the overall effect size measures of the effect of exercise on subjective function in patients with AC (k = 5, Q(4) = 20.15, *p* < 0.001, I^2^ = 80%). Under the random-effects model, the overall effect of exercise on subjective function was statistically significant (SMD = 3.15, 95% CIs = 2.06 to 4.24), indicating that exercise improved subjective function in patients with AC compared to pre-exercise (large effect size).

**Figure 8:**
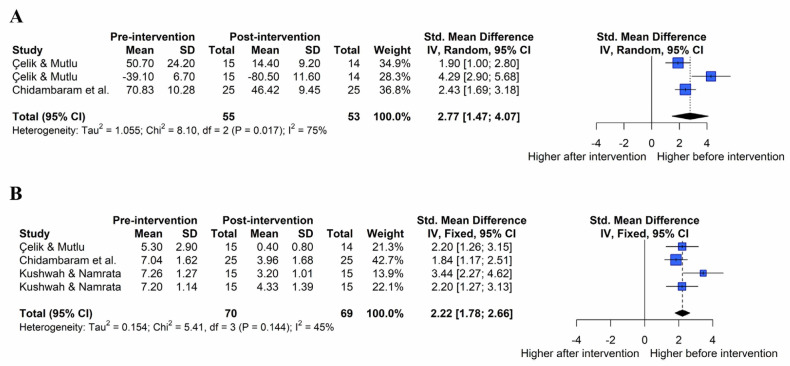
Effect of a combination of exercise and joint mobilization on patient-oriented outcome in forest plots of the meta-analysis. Subjective function (**A**) and pain (**B**) [22,35,36].

The effect of the combination of exercise and joint mobilization on subjective function was assessed (Figure 8A; k = 3, Q(2) = 8.10, p = 0.017, I^2^ = 75%). Under the random-effects model, the overall effect of a combination of exercise and joint mobilization on subjective function was statistically significant (SMD = 2.22, 95% CIs = 1.47 to 4.07), indicating that a combination of the two interventions improved subjective function in patients with AC compared to pre-intervention (large effect size).

#### 3.4.7. Pain

Figure 7B shows the overall effect size measures of the effect of exercise on pain in patients with AC (k = 4, Q(3) = 42.28, *p* < 0.001, I^2^ = 93%). Under the random-effects model, the overall effect of exercise on pain was statistically significant (SMD = 4.13, 95% CIs = 1.86 to 6.41), indicating that exercise improved pain in patients with AC compared with pre-exercise (large effect size).

The effect of the combination of exercise and joint mobilization on pain was assessed (Figure 8B; k = 4, Q(3) = 5.41, *p* = 0.144, I^2^ = 45%). Under the fixed-effects model, the overall effect of a combination of exercise and joint mobilization on pain was statistically significant (SMD = 2.22, 95% CIs = 1.78 to 2.66), indicating that a combination of the two interventions improved pain in patients with AC compared to pre-intervention (large effect size).

## 4. Discussion

This study aimed to suggest an integrated conclusion on the effect of exercise and exercise with joint mobilization on patients with AC by performing a systematic review and meta-analysis. Nine studies were analyzed, and, as the overall effect size was >0.8, we concluded that exercise and exercise combined with mobilization could be effective interventions to recover function and ROM and relieve pain. In addition, a greater effect on shoulder ROM recovery was shown when joint mobilization was added to exercise. On the contrary, subjective function (shoulder pain and disability index (SPADI), disabilities of arm, shoulder, and hand (DASH), and constant shoulder score) and pain were further improved with exercise without joint mobilization. The results are as follows.

### 4.1. Effect on Shoulder ROM

AC causes a reduction in external shoulder rotation ROM, which indicates that the inter-rotator cuff and the coracohumeral ligament are aggravated; therefore, exercises including joint stretching should be applied [37]. In this review, a study [30] that applied the CHL stretch, while grabbing a dowel with adducted and hyperextended humerus and forearm supinated in the side-lying position on the unaffected arm, showed a significant effect size (0.87). In particular, previous studies [22,29] have shown that the effect was greater when strength exercises were combined. These results contradict those of previous studies [38], which inferred that strength exercises were unnecessary. Alternatively, the necessity of concurrent strength exercises to recover external rotation ROM was highlighted. In addition, a previous study [22] showed that the addition of glenohumeral joint distraction, caudal glide, posterior glide, and anterior glide to scapulothoracic and rotator cuff muscular strength exercise were ideal intervention methods for restoring external shoulder rotation and abduction ROM (grade I or II rhythmic oscillations were used in the first 2 weeks, and grade III and IV oscillation techniques were used in the following week.) These effects are thought to be due to not only securing PROM through joint mobilization but also the recovery of the dynamic musculoskeletal system through active muscle strength exercise. However, active-ROM and passive-ROM restriction of the glenohumeral joint due to AC is often multidirectional in practice [11,12]. Although this review showed that all three axes of shoulder ROM improved, it is difficult for this to be explained by a functional anatomical viewpoint based on this evidence. Since studies on connective tissues involved in ROM other than external rotation remain scarce, follow-up studies are deemed necessary.

### 4.2. Effect on Shoulder Subjective Function and Pain

If the cost-effectiveness of AC patients is considered, the importance of autonomy may increase; in fact, several studies have reported that participants who exercised showed better results regardless of whether other treatments were combined [39]. A previous study reviewing AC [5] showed that the recovery of AC yields better subjective results than objective indicators. In this study, exercise without joint mobilization had a greater effect on subjective function (SPADI, DASH, and constant shoulder score) and pain relief. Unlike passive exercises, such as mobilization by a therapist, the patient could recognize pain-free ROM well through active exercise, and the patient was self-aware and actively created motion by contracting and relaxing the muscles, thus restoring proprioception and kinesthesia. Therefore, these results could be explained by the feeling of being liberated from discomfort in daily life. In fact, AC patients in the ‘frozen’ phase had satisfactory results after applying stretching in the direction of external rotation, internal rotation, and horizontal adduction with tolerable intensity [40]. However, in a state where pain remains due to AC, the stretching intensity should be adjusted according to the degree of pain, and, for this, the stimulation sensitivity of each patient should be considered [39]. Low sensitivity to stimuli means that stiffness of the joint is felt relatively more strongly than pain, so it responds better to more intense movements or stretching. For AC patients with this type, more aggressive exercise interventions could be implemented so there is a possibility of better recovery. However, in the case of high stimulation sensitivity, since pain is felt more strongly than stiffness, exercise intervention is considered to be performed cautiously, and there are no guidelines based on solid evidence. Therefore, ultrasound, iontophoresis, and phonophoresis may be recommended, and it has been found that when these patients received ultrasound treatment, ROM improved, but there was no improvement in pain or function [41,42]. Therefore, a cognitive improvement program that can lower stimuli sensitivity should be developed and applied so that patients can better accept exercise interventions.

### 4.3. Implications of Virtual Reality Training and Coracohumeral Ligament Stretching

High adherence should be induced to maximize the efficiency of the prescribed exercise program for AC. In one study [40], participants were instructed to exercise five times a day but actually showed compliance with an average of two times a day. Hence, a sufficient dose of exercise intervention could be applied when patient interest is aroused. As such, virtual reality (VR) training could be an enjoyable and interesting approach for AC patients. However, analyzing the research of applying VR training [33] in this review showed that the effect sizes of internal rotation, external rotation, and abduction were small compared to those in other studies and were not statistically significant. Hence, developing and modifying the existing VR program is necessary to pique the interest of patients with AC and drive better ROM recovery. Furthermore, when coracohumeral ligament stretching, which was found to improve external shoulder rotation ROM [37,43], was applied, the effect size for the recovery of shoulder flexion and abduction ROM was not significant [30]. Since shoulder flexion and abduction play an essential role in picking up objects overhead or enjoying leisure activities requiring considerable overhead movement (e.g., racquet sports), joint stretching in various directions besides the CHL should be applied to AC patients. Accordingly, a study that performed stretching of the anterior, posterior, and antero-inferior capsules [36] was analyzed, but the effect size was not significant in shoulder flexion ROM recovery. If restoration of the shoulder flexion ROM is given priority, the proportion of exercises not directly related to the shoulder, such as sit-to-stand, must be reduced, and, in addition to the joint capsule, treatment for shoulder flexor muscles such as the anterior deltoid, pectoralis major, serratus anterior, and upper and lower trapezius muscles [44] seems necessary.

### 4.4. Overall Implications and Limitations

This study aimed to create a framework for a protocol that enables efficient treatment by selectively applying exercises and joint mobilization, which can be used conservatively. 

Exercise does not generally incur any cost and for most individuals can be easily practiced in daily life. This review indicates the significant effectiveness of exercise for patients with AC. In addition, by comprehensively investigating the ROM of the shoulders of patients with AC and subjective indices, data for an evidence-based exercise program were presented. Based on the results of this review, interventions including exercise should be preferentially performed, particularly for pain relief. However, they can be more effective in improving ROM when combined with joint mobilization by clinicians. However, a limitation of this study is that it is difficult to analyze subgroups according to the type of exercise performed, as all types of exercises are based on a certain protocol, and a comparative study on the effect according to the stage of frozen shoulder and the presence or absence of comorbidities is required. In addition, as shown from the quality assessment of the studies presented in Table 3, information on adherence to exercise intervention was not found in the studies included in this review. Therefore, further research is required to determine the degree of exercise program practice in patients with AC. In addition, there are several interventions, such as injection and transcutaneous electric nerve stimulation, in addition to exercise, for the treatment of AC. However, since this review only investigated the net effect of exercise, it could not explain the case when interventions other than exercise and joint mobilization were additionally applied. Furthermore, since the focus was on presenting the integrated viewpoint, low-score studies were also included in the analysis in the risk of bias evaluation process, and blindness of individual studies was also not considered; therefore, caution is required when interpreting the results. In addition, since the literature published before 2000 was not included in the analysis, the possibility of bias in the process of deriving the pooled effect size cannot be excluded. Finally, since the literature search protocol of this study was conducted in an unregistered state, it is suggested that follow-up research proceeds after registering the research protocol with an institution such as PROSPERO.

Dogru, Basaran, and Sarpel [41] reported that when patients with AC received ultrasound treatment, their ROM improved, but there was no pain or functional improvement. Moreover, Jewell, Riddle, and Thacker [42] reported that the ion transfer-phonophoresis method and ultrasonic therapy massage reduced the improvement in function in 19–32% of patients. Therefore, it is suggested that interventions other than exercise can vary, but exercise and joint mobilization should be included in the rehabilitation of patients with AC. Therefore, as future comparisons between various intervention strategies are required, individual studies should be conducted to gather sufficient evidence. In addition, the characteristics of the participants, such as racial differences and comorbidities, were not considered. An epidemiological study showed that the Black/African American and Hispanic/Latino populations are more likely to develop AC [45]. In addition, as AC occurs more frequently in patients with cerebrovascular accidents, myocardial infarction, and diabetes mellitus [46,47,48], it is necessary to consider the characteristics of these patients. Regarding ROM, many studies in our meta-analysis did not specify the measurement method; hence, they were integrated and analyzed. However, the difference between active and passive ROM was reported by Jellad et al. [32]; hence, caution is warranted when interpreting the results. In addition, the progression stage of AC was not considered, and the exercise protocol and dose of mobilization were not specified; thus, considering these limitations, analysis was not possible.

## 5. Conclusions

In this study, we aimed to comprehensively investigate the effects of exercise combined with joint mobilization, a conservative rehabilitation method, on ROM and subjective symptom recovery in patients with frozen shoulder. The following conclusions were drawn (by comparing the differences) to provide evidence for the selection of an effective intervention method. First, exercise and exercise with joint mobilization both had significant effects on the recovery of shoulder flexion, extension, internal rotation, external rotation, abduction ROM, pain, and subjective function improvement in patients with AC. Second, our results indicate that, to recover ROM, the effect increases only when joint motion therapy is applied to exercise therapy. To eliminate subjective discomfort in daily life, including pain, it is desirable to configure the rehabilitation protocol to include only exercise. Therefore, it is appropriate to construct an AC rehabilitation exercise program by identifying all patient symptoms and objective indicators and adjusting the weight of exercise and joint mobilization to reflect patient-specific needs.

## Figures and Tables

**Table 1:** Characteristics of studies with exercise intervention.

Author (year)	Study Design	Participants (n, Age)	Inclusion Criteria of Participants	Exclusion Criteria of Participants	Exercise Intervention	Outcome Measurement	Major Findings
Anjum et al. (2020) [29]	RCT	PT (n = 26, 41.12) PT + IASI (n = 26, 44.46)	Diagnosed idiopathic frozen shoulder of <6 month duration, age between 18 and 65 years, nontraumatic stiff shoulder, non-diabetic state, and restriction of active and passive motion >30° in two or more planes.	Systemic inflammatory joint disease, skeletally immature patients, contraindications to joint distension including allergy to local anesthetic, and shoulder abnormality detected on plain X-ray	Stretching, pendulum exercises, active-assisted exercises. isometric, concentric strengthening exercises	Index (SPADI), pain (VAS), ROM (flexion, abduction, external rotation) muscular power (supraspinatus, infraspinatus, subscapularis)	Range of shoulder flexion, abduction, external rotation, and SPADI significantly improved in PT + IASI compared to PT
Bhavana and Soni (2017) [30]	Pre-post	n = 16, 40–80	Duration of complaints of >3 months, age 40–80 years old, spontaneous onset of the painful stiff shoulder, movement impairment in 1 or more of 3 movement directions, loss of active and passive shoulder motion with external rotation restriction of <50, an extension of <30, and adduction <30	Not described	Stretching of coracohumeral ligament	ROM (flexion, extension, external rotation, abduction, adduction)	Passive range of motion was significantly improved following stretching of coracohumeral ligament
Gupta et al. (2019) [31]	CER	supervised OT (n = 16, 40–60) Home based OT (n = 19, 40–60)	AC with diabetes mellitus, with age group 40–60	History of any life-threatening disease or history of any musculoskeletal injury, traumatic injury, or shoulder pain due to fracture or any other pathology in past 6 months	Occupational therapy	Index (SPADI) ROM (flexion, extension, internal rotation, abduction	Both groups showed no significant difference in any of the variables.
Jellad et al. (2020) [32]	RCT	IAD-PT (n = 34, 55.7 ± 9.8) PT, IAD (n = 46, 55.1 ± 7.7) PT (n = 42, 55 ± 10.4)	Primitive AC of the shoulder	AC secondary to trauma, operation, hypothyroidism, stroke, rotator cuff lesion. Tendon rupture. Those who had a previous IAD, and those who had physical therapy or intra-articular corticosteroid injection in the last three months	Stretching, pendulum exercises passive, active-assisted exercises	Index (DASH), Pain (VAS), PROM, AROM (FLEX, ER, ABD)	IAD followed by PT is more beneficial than IAD preceded by PT in terms of upper extremity function
Lee et al. (2016) [33]	Pre-post	n = 16, 58.6	20 < age, not received PT, clinically confirmed to have frozen shoulders	History of a proximal humeral fracture or dislocation of glenohumeral joint, received hyaluronic acid injection in the shoulder, experienced cervical radiculopathy or diagnosed with shoulder degenerative joint diseases, at the final stage of a malignant disease, pregnant.	Stretching, shoulder, core strengthening	ROM, strength (FLEX, ER, IR, ABD)	Task performance, motor indices, and the clinical assessments indicated significant improvement for most of the assessed items. Task performance effectively predicted the results of several clinical assessment items.
Shishir et al. (2013) [34]	Pre-post	n = 36, 33–73	Symptom of >4 weeks and <6 months, 25% limited active, passive range of ABD, ER compared with the other shoulder, minimum follow-up 2 years	Pain of <4, osteopenia, received intra-articular injection or prior PT before start of the protocol, limitation of movement in one plane only, pain originated from acromioclavicular joint, presenting with frozen shoulder secondary to other disease, osteoarthritis, rotator cuff tear, cervical spine disease, trauma, inflammation	Shoulder Accelerated Rehabilitation Protocol	Constant score, pain (VAS), ROM (ER, ABD)	The mean constant shoulder score at the start of protocol was 26.69 (SD-8.522), which improved to 98.58 (SD-2.892) at 15 months. Pain score at the start of the protocol was 7.14 (SD-1.222) which improved at 18 months

Abbreviations: RCT, randomized controlled trial; PT, physiotherapy; IASI, intra-articular steroid injection; SPADI, shoulder pain and disability index; VAS, visual analog scale; ROM, range of motion; CER, comparative experimental research; OT, occupational therapy; AC, adhesive capsulitis.

**Table 2:** Characteristics of studies with exercise intervention and joint mobilization.

Author (Year)	Study Design	Participants (n, age)	Inclusion Criteria of Participants	Exclusion Criteria of Participants	Exercise & Mobilization	Outcome Variables	Major Findings
Chidambaram et al. (2020) [35]	RCT	MWM (n = 25, 40–70) MWM + stretching (n = 25, 40–70)	AC at stage 1 and 2 (David J Magee) were included between the age groups of 40 to 70 years with minimum 90 degrees of shoulder FLEX and ABD.	Possess malignancy, neck pain with radiculopathy and recent shoulder injuries	Sleeper stretching (only for MWM + stretching group), pendular exercises, finger ladder exercises and active ROM and glide to increase FLEX, IR, ER	Index (SPADI), Pain (NPRS) ROM (FLEX, IR, ER)	The group which received sleeper’s stretch with MWM shows high significant in improving range of motion and pain.
Çelik and Mutlu (2016) [22]	RCT	Stretching (n = 12, 54.8 ± 6.4) Stretching +mobilization (n = 14, 54.2 ± 7.9)	Range of FLEX, ER, ABD less than 50% in comparison to uninvolved shoulder, normal radiographic results (anteroposterior and lateral views), complaint of >months	Cervical radiculopathy and radiating pain from wrist or hand, diabetes, thoracic outlet syndrome, rheumatological disorders, fractures, or tumors of either upper extremity, neurological disorders that cause muscle weakness in the shoulder corticosteroid injections in the affected shoulder within the previous 4 weeks, rotator cuff tears	Cyclic stretching, strengthening exercises for the scapulothoracic and rotator cuff muscles, and caudal, anterior, posterior glide of glenohumeral joint (stretching +mobilization group)	Index (DASH, Constant score) Pain (VAS), PROM (FLEX, ER, IR, ABD)	Significant increases in ABD, ER and constant score at the 1-year follow-up in the stretching +mobilization, whereas stretching group did not show significant changes.
Kushwah and Namrata (2018) [36]	RCT	Capsular stretch (n = 15, 40–60) Capsular stretch +ART (n = 15, 40–60	Painful stiff shoulder of >3 months, age between 40–60, limited ROM compared to non-involved side, inability to lie on affected shoulder, primary idiopathic periarthritis shoulder	History of surgery on the shoulder, rotator cuff rupture, pain related to trauma, fracture of the shoulder complex, osteoarthritis, or signs of bony damage, inflammatory diseases (i.e., rheumatoid arthritis)	ABD, ER exercise (capsular stretch + ART group alone) Sit or stand exercise and stretch of anterior, posterior, antero-inferior capsule	Pain (VAS), ROM (FLEX, EXT, ER, IR, ABD)	Significant improvement in both groups for VAS and ROM. Significant difference in effect of capsular stretch +ART with conventional therapy than capsular stretch only

Abbreviations: RCT, randomized controlled trial; MWM, movement with mobilization; AC, adhesive capsulitis; FLEX, flexion; EXT, extension; IR, internal rotation; ER, external rotation; ABD, abduction; SPADI, shoulder pain and disability index; NPRS, Numerical Pain Rating Scale; DASH, disabilities of arm, shoulder, and hand questionnaire; VAS, visual analog scale; PROM, passive range of motion; ART, active release technique.

**Table 3:** Quality assessment of studies.

NIH Study Quality Assessment Tools	Anjum et al. (2020) [29]	Bhavana and Soni (2017) [30]	Çelik and Mutlu (2016) [22]	Chidambaram et al. (2020) [35]	Gupta et al. (2019) [31]	Jellad et al. (2020) [32]	Kushwah and Namrata (2018) [36]	Lee et al. (2016) [33]	Shishir et al. (2013) [34]
1. Was the study described as randomized, a randomized trial, a randomized clinical trial, or an RCT? Was the method of randomization adequate?	Yes	No	Yes	Yes	No	Yes	Yes	No	No
2. Was the study described as a controlled trial? Was the control group matched on relevant variables (age, gender, education, disorder)?	Yes	No	Yes	Yes	Yes	Yes	Yes	No	No
3. Was the overall dropout rate from the study at endpoint 20% or lower of the number allocated to the intervention?	Yes	Yes	Yes	Yes	Yes	Yes	Yes	Yes	Yes
4. Was the differential dropout rate (between groups) at endpoint 15 percentage points or lower?	Yes	CD	Yes	Yes	Yes	Yes	Yes	Yes	Yes
5. Was there high adherence to the intervention protocols for each treatment group? (Defined as 75 % attendance or more)	CD	CD	CD	CD	CD	CD	CD	CD	CD
6. Were other interventions avoided or similar in the groups?	Yes	Yes	Yes	Yes	Yes	Yes	Yes	Yes	Yes
7. Were outcomes assessed using valid and reliable measures?	Yes	Yes	Yes	Yes	Yes	Yes	Yes	Yes	Yes
8. Were outcomes measured consistently across all study participants?	Yes	Yes	No	Yes	Yes	No	Yes	Yes	Yes
9. Did the authors report that the sample size was sufficiently large to be able to detect a difference in the main outcome between groups with at least 80% power?	Yes	No	Yes	Yes	Yes	Yes	Yes	No	Yes
10. Were outcomes reported or subgroups analyzed prespecified (i.e., identified before analyses were conducted)?	Yes	Yes	Yes	Yes	Yes	Yes	Yes	Yes	Yes
11. For RCTs, were all randomized participants analyzed in the group to which they were originally assigned? For controlled studies, was a recognized statistical method employed?	Yes	Yes	Yes	Yes	Yes	Yes	Yes	Yes	Yes
Score (%)	10 (91)	6 (55)	9 (82)	10 (91)	10 (91)	9 (82)	10 (91)	7 (64)	8 (73)
Level of evidence	1	2	1	1	1	1	1	2	1

Abbreviations: CD, cannot decide.

## Data Availability

The data can be directed to the corresponding author.
